# S08-2: Challenges of cooperation between urban planning, sustainable mobility, sport, and public health for effective HEPA policies - lessons from Slovenia

**DOI:** 10.1093/eurpub/ckae114.233

**Published:** 2024-09-26

**Authors:** Ina Šuklje Erjavec, Jana Kozamernik, Vita Žlender

**Affiliations:** Urban Planning Institute of the Republic of Slovenia; Urban Planning Institute of the Republic of Slovenia; Urban Planning Institute of the Republic of Slovenia

## Abstract

**Purpose:**

In accordance with state or federal legislation in each country, local governments have public health related responsibilities, to protect and promote health in the populations. Due to changes in the natural & built environments, work characteristics, sedentarism became an important public health issue. Therefore, in the last 15-20 years, physical activity (PA) became more and more prominent in the preoccupations of national and local governments, with strategies, policies and programs to promote PA being issued, adopted and some implemented and evaluated.

The aim of this study is to explore the potential gap between local governments’ PA promotion related goals (as stated in their official PA related strategies or policies) and the actual PA projects or programs implemented at local level targeting diverse populations subgroups.

**Methods:**

We used the CAPLA-Santé local policy assessment tool to describe the PA related strategies or policies in 5 selected municipalities from Finland, France, Germany, Japan and Romania. This presentation focuses on the respective target audiences of PA policy (CAPLA-Santé question 11) and examples of concrete actions in the form of programmes, interventions, or structuring initiatives (question 14).

**Results:**

In each of the 5 selected municipalities, the local PA related strategies or policies include diverse populations subgroups such as: pre-school, children/adolescents, students, women, people in care facilities/patients suffering from chronic diseases, migrants, disabled individuals, seniors etc. However, from the examples of good practice reported, for all municipalities, only some of these subgroups (i.e. seniors, patients suffering from chronic diseases, women) are targeted in the PA related programs, interventions, or initiatives.

**Conclusions:**

Even though inclusion in public health / PA promotion is at declarative / goals level on the agenda for all analyzed municipalities, in practice, only a few population subgroups benefit from local dedicated PA promotion initiatives. Reasons for this and potential ways to improve the current status quo are discussed.

**Funding source:**

European Commission, Erasmus Plus programme, 613568-EPP-1-2019-1-RO-SPO-SSCP

